# Iron Accumulates in Retinal Vascular Endothelial Cells But Has Minimal Retinal Penetration After IP Iron Dextran Injection in Mice

**DOI:** 10.1167/iovs.19-28250

**Published:** 2019-10

**Authors:** Wanting Shu, Bailey H. Baumann, Ying Song, Yingrui Liu, Xingwei Wu, Joshua L. Dunaief

**Affiliations:** 1 Department of Ophthalmology, Shanghai General Hospital, Shanghai Jiao Tong University School of Medicine, Shanghai Key Laboratory of Ocular Fundus Diseases, Shanghai Engineering Center for Visual Science and Photomedicine, Shanghai, China; 2 F.M. Kirby Center for Molecular Ophthalmology, Scheie Eye Institute, Perelman School of Medicine at the University of Pennsylvania, Philadelphia, Pennsylvania, United States; 3 Department of Ophthalmology, The Second Hospital of Jilin University, Changchun, Jilin, China

**Keywords:** iron, retinal vascular endothelial cells, ferritin, ferroportin, hepcidin

## Abstract

**Purpose:**

Iron supplementation therapy is used for iron-deficiency anemia but has been associated with macular degeneration in a 43-year-old patient. Iron entry into the neurosensory retina (NSR) can be toxic. It is important to determine conditions under which serum iron might cross the blood retinal barrier (BRB) into the NSR. Herein, an established mouse model of systemic iron overload using high-dose intraperitoneal iron dextran (IP FeDex) was studied. In addition, because the NSR expresses the iron regulatory hormone hepcidin, which could limit iron influx into the NSR, we gave retina-specific hepcidin knockout (RS-*Hepc*KO) mice IP FeDex to test this possibility.

**Methods:**

Wild-type (WT) and RS-*Hepc*KO mice were given IP FeDex. In vivo retina imaging was performed. Blood and tissues were analyzed for iron levels. Quantitative PCR was used to measure levels of mRNAs encoding iron regulatory and photoreceptor-specific genes. Ferritin and albumin were localized in the retina by immunofluorescence.

**Results:**

IP FeDex in both WT and RS-*Hepc*KO mice induced high levels of iron in the liver, serum, retinal vascular endothelial cells (rVECs), and RPE, but not the NSR. The BRB remained intact. Retinal degeneration did not occur.

**Conclusions:**

Following injection of high-dose IP FeDex, iron accumulated in the BRB, but not the NSR. Thus, the BRB can shield the NSR from iron delivered in this manner. This ability is not dependent on NSR hepcidin production.

Iron is fundamental for life, but excess iron can be toxic within the Fenton reaction, which produces highly reactive hydroxyl free radicals. In the retina, photoreceptors (PRs) are especially vulnerable to oxidative damage because of photo-oxidation and high retinal oxygen tension. There is high concentration of easily oxidized polyunsaturated fatty acids in PR outer segments, which are phagocytosed by RPE cells each day. Therefore, iron must be tightly regulated to protect the PRs and RPE cells from oxidative stress.

Retinal iron toxicity has been implicated in the pathophysiology of retinal degenerative diseases, including AMD,^1^–^3^ and aceruloplasminemia.^4^,^5^ In addition, retinal degeneration has been described in diseases that involve serum iron overload including hemochromatosis,^6^ as well as in several mouse models of hereditary retinal iron overload. Mice with double knockout of ferroxidases ceruloplasmin and hephaestin (*Cp*/*Heph* DKO),^7^ knockout of the iron regulatory hormone hepcidin (Hepc),^8^ or its secreted inducer, bone morphogenic protein 6 (Bmp6),^9^,^10^ or the Bmp6 coreceptor hemojuvelin^11^ have elevated iron levels in the serum, neurosensory retina (NSR), and RPE, resulting in diffuse RPE autofluorescence/hypertrophy, photoreceptor degeneration, and, occasionally, focal subretinal neovascularization.

Compared with hereditary iron overload mouse models, systemic iron overload induced by dietary or injected iron induce much milder phenotypes. Wild-type (WT) mice fed a high iron diet for 10 months had increased iron levels in the RPE but not NSR, and did not exhibit NSR or RPE degenerative changes.^12^ WT mice exposed to 10 months of high systemic iron by intravenous (IV) injections of iron-sucrose had increased iron levels in the RPE and choroid, and mild increases in the NSR, with focal RPE hypertrophy and Bruch's membrane thickening.^9^,^13^

The reason why the high iron diet and IV iron-sucrose successfully induced systemic iron overload but failed to replicate the severe retinal phenotype seen in the KO mouse models is not yet understood. One possible explanation is that although dietary or IV iron administration increased RPE iron, they only minimally increased NSR iron. In contrast, the genetic models increased both NSR and RPE iron. This hypothesis is supported by observations that iron can oxidize photoreceptor outer segments when injected into the eye, and this is toxic to both the PRs and RPE.^14^–^16^ Thus, it is important to determine whether there are any conditions under which exogenously administered iron penetrates the blood retinal barrier (BRB), and to understand how the NSR regulates iron influx.

The BRB is composed of two components, an inner and an outer barrier. The inner BRB is formed by the tight junctions between the retinal vascular endothelial cells (rVECs) with support from pericytes and Müller cell endfeet.^17^,^18^ The outer BRB is composed of the tight junctions between the RPE, shielding the NSR from the choriocapillaris. Ferroportin (Fpn), the only known cellular iron exporter,^19^ is localized to the abluminal membrane of the rVECs and basolateral RPE,^20^ suggesting that Fpn may transfer iron from the rVECs into the NSR, and from the RPE into the choriocapillaris. Supporting this assertion, conditional knockout of Fpn in the rVECs leads to elevated ferritin levels in the rVECs and diminished iron levels in the NSR.^21^ Regulation of Fpn on the abluminal membrane may protect the NSR from iron overload in the iron supplementation models.

In contrast, Fpn regulation does not appear to protect the retina from iron overload in the KO models, most of which impair the iron regulatory hormone hepcidin (Hepc). In the gut, macrophages, and reticuloendothelial system, secreted Hepc triggers degradation of Fpn, limiting cellular iron export.^22^ Similarly, Hepc administration triggers a reduction in Fpn levels and diminished iron export from cultured rVECs.^8^ Consistent with this, AAV-Hepc injection into the mouse retina leads to increased rVEC ferritin,^20^ suggesting that Hepc may prevent Fpn-mediated iron export from the abluminal membrane of the rVECs into the NSR.

Parenteral iron therapy for iron-deficiency anemia increases hemoglobin levels more rapidly than oral iron, because it circumvents the limitation of intestinal iron absorption,^23^ but it may increase the risk of iron-induced retinopathy. A 43-year-old woman with iron-deficiency anemia developed retinal drusen within 11 months of therapy with iron-sucrose, suggesting that IV iron therapy may have caused retinal iron accumulation that promoted early AMD.^13^ Hence, it is important to investigate secondary iron overload mouse models using different routes of administration on young and aged mice for various periods of time to assess the retinal safety of parenteral iron therapy and the mechanisms and limitations of Fpn/Hepc-mediated retinal iron regulation across the BRB.

In the current study, we used a secondary iron overload mouse model established through intraperitoneal (IP) iron dextran (FeDex) injection to young adult mice, which led to systemic iron overload and cardiac dysfunction.^24^ Young adult and aged mice were exposed to high systemic iron levels through IP FeDex injection in the short or long term. Retinal iron levels and potential retinal degeneration were assessed. Mechanisms of retinal iron regulation by rVECs were investigated using retina-specific *Hepc*KO mice.

## Materials and Methods

### Animals

Experimental procedures were performed in accordance with the ARVO Statement for the Use of Animals in Ophthalmic and Vision Research. All protocols were approved by the animal care review board of the University of Pennsylvania. Adult male WT C57BL/6J mice (Stock No. 000664) aged 2 months were purchased from The Jackson Laboratory (Bar Harbor, ME, USA). Adult male WT C57BL/6J mice aged 18 months were provided by the National Institute on Aging. Retina-specific *Hepc*KO mice were created as previously described,^25^ and aged to 4 months old for the study. Retina-specific *Hepc*KO mice had the genotype Hepc^flox/flox^, mRx-Cre^+^, and the control mice had the genotype Hepc^+/+^, mRx-Cre^+^. Both experimental and control mice were on a C57BL/6J background and both males and females were used in this study. All mice were fed a standard laboratory diet and given free access to water; they were maintained in a temperature-controlled room at 21 to 23 °C under dim cyclic light (12 h:12 h light-dark cycle) during the experiments.

### Assignment of Experimental Groups

**Figure 1:**
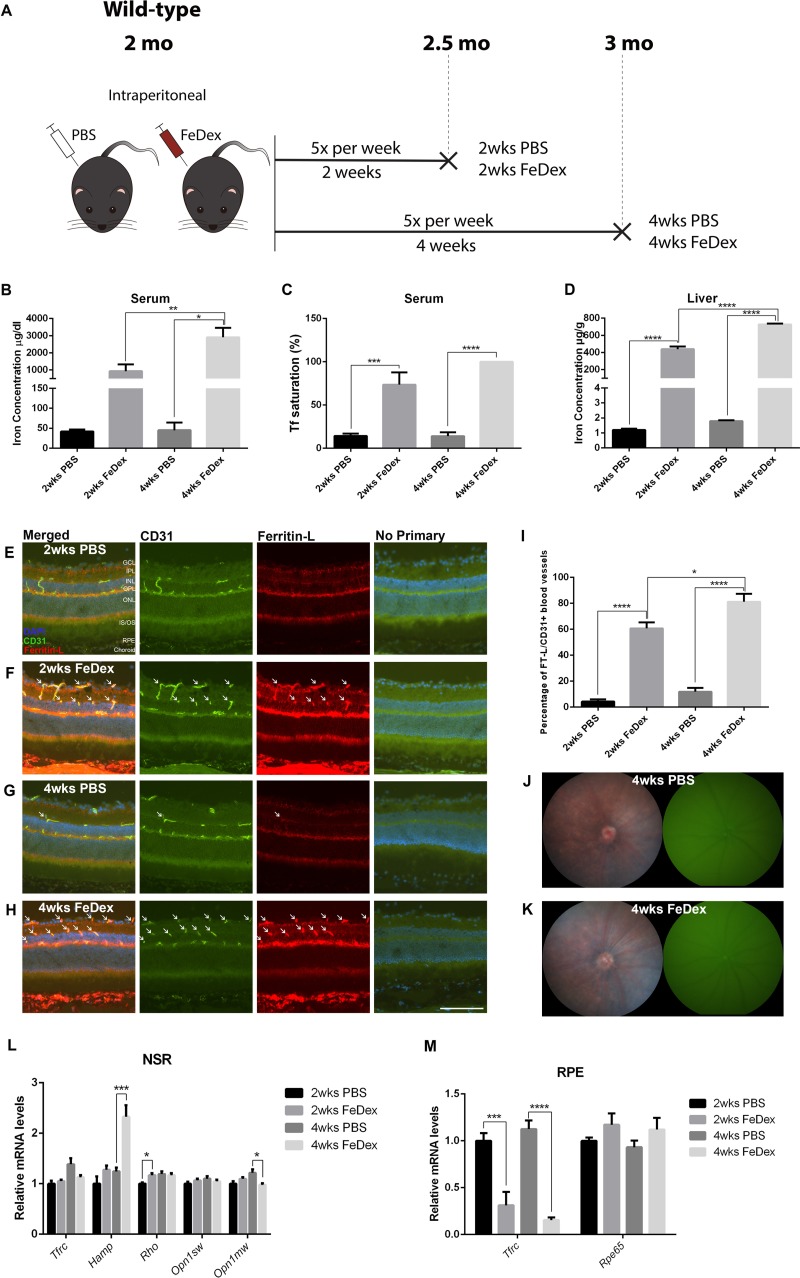
One month of IP FeDex does not increase NSR iron but causes elevation of hepcidin and iron-trapping in the retinal vasculature. (A) Schematic illustration of the experimental design of the first cohort, 2-month-old WT mice. Experimental mice were IP injected with FeDex for 2 or 4 weeks, control mice were injected with PBS. Each group of mice was killed 24 hours after the last injection. (B) Graph of serum iron concentration of each group. (C) Tf saturation in the serum. (D) Liver iron concentration of each group. (E–H) Ferritin-L retinal immunostaining. There was increased Ferritin-L labeling within the rVECs (identified by the colabeling with CD31) in mice receiving either 2 and 4 weeks of FeDex injections (white arrows, F and H) compared with PBS-injected controls (E and G). (I) Quantification of the percentage of Ferritin-L+, CD31+ blood vessels in the retinas of each group. (J and K) Representative in vivo color fundus and green autofluorescence images of mice receiving 4 weeks FeDex injections (K) and matched controls (J), showing no gross retinal phenotype or autofluorescence in either group. (L) Graph of relative mRNA levels of Tfrc, Hamp, Rho, Opn1sw, and Opn1mw determined by qPCR in NSR. (M) Graph of relative mRNA levels of Tfrc and Rpe65 determined by qPCR in RPE. Statistical analyses were performed using 1-way ANOVA with post hoc pairwise comparisons using the Bonferroni method. Error bars indicate ± SEM, *P < 0.05, **P < 0.01, ***P < 0.001, ****P < 0.0001. Scale bars: 50 μm. n = 4–5 per group. GCL, ganglion cell layer; IPL, inner plexiform layer; INL, inner nuclear layer; OPL, outer plexiform layer; ONL, outer nuclear layer; IS/OS, inner and outer segments.

**Figure 2:**
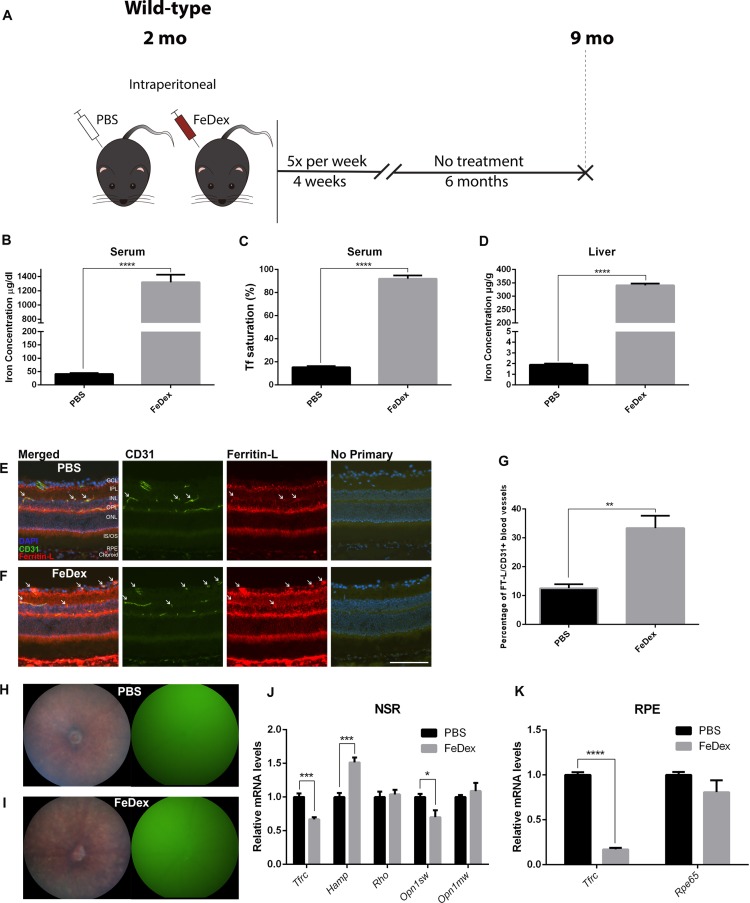
Seven months of exposure to high systemic iron through IP FeDex causes mild increase in NSR iron, hepcidin, and mild reduction in cone opsin. (A) Schematic illustration of the experimental design of the second cohort. Two-month-old WT mice were IP injected with FeDex or PBS for 4 weeks, and aged for 6 months until euthanized. (B) Graph of serum iron concentration of FeDex-injected mice and control. (C) Serum Tf saturation of each group. (D) Graph of liver iron concentrations. (E, F) Ferritin-L immunostaining in the cryosections of the retinas. There was increased Ferritin-L labeling within the CD31+ rVECs (white arrows) of the FeDex group (F) compared with control (E). (G) Quantification of the percentage of Ferritin-L+, CD31+ blood vessels in the retinas. (H, I) Representative in vivo color fundus photos and green fluorescence images of FeDex-injected mice (I) and PBS-injected mice (H). (J) Graph of relative mRNA levels of Tfrc, Hamp, Rho, Opn1sw, and Opn1mw determined by qPCR in NSR of each group. (K) Graph of relative mRNA levels of Tfrc and Rpe65 determined by qPCR in RPE of each group. Statistical analyses were performed using Student's two-group, two-sided t-test. Error bars indicate ± SEM, **P < 0.01, ***P < 0.001, ****P < 0.0001. Scale bars: 50 μm. n = 5 per group.

**Figure 3:**
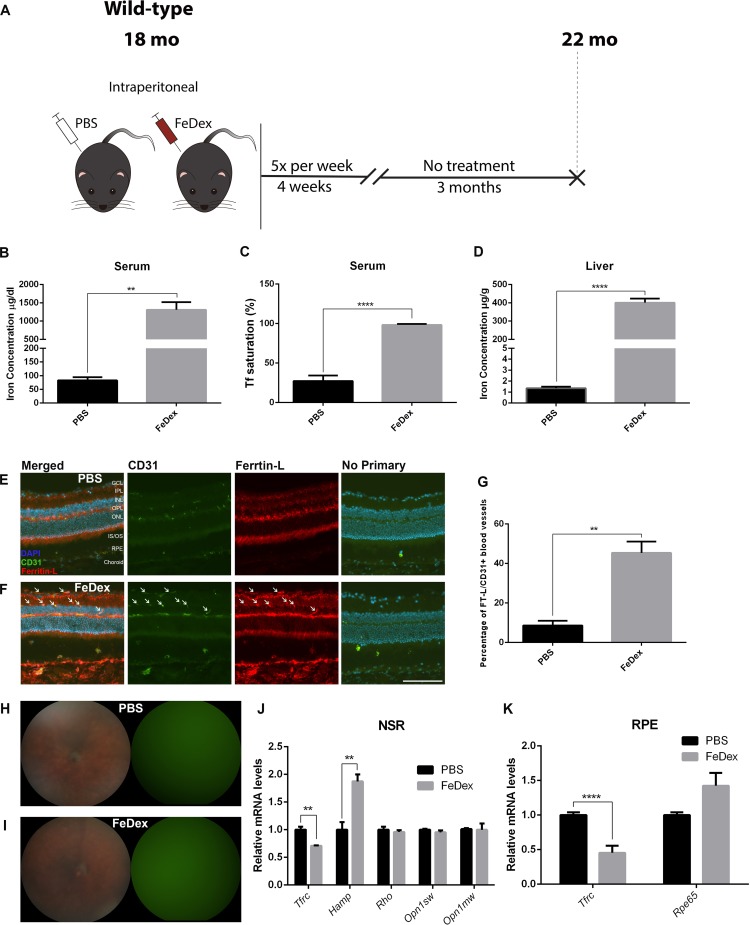
Four months of exposure to high systemic iron in 18-month-old mice through IP FeDex mildly increases NSR iron and hepcidin, but does not cause photoreceptor degeneration. (A) Schematic illustration of the experimental design of the third cohort. 18-month-old WT mice were IP injected with FeDex or PBS for 4 weeks, and aged for 3 months until euthanization. (B) Graph of serum iron concentration of each group. (C) Serum Tf saturation. (D) Graph of liver iron concentration. (E and F) Ferritin-L immunostaining in the cryosections of the retinas. There was increased Ferritin-L labeling within the CD31+ rVECs (white arrows) of FeDex group (F) compared with control (E). (G) Quantification of the percentage of Ferritin-L+, CD31+ blood vessels in the retinas. (H, I) Representative in vivo color fundus photos and green fluorescence images of FeDex-injected mice (I) and PBS-injected mice (H). (J) Graph of relative mRNA levels of Tfrc, Hamp, Rho, Opn1sw, and Opn1mw determined by qPCR in NSR of each group. (K) Graph of relative mRNA levels of Tfrc and Rpe65 determined by qPCR in RPE of each group. Statistical analyses were performed using Student's two-group, two-sided t-test. Error bars indicate ± SEM, **P < 0.01, ****P < 0.0001. Scale bars: 50 μm. n = 3–4 per group.

Iron dextran (FeDex, ferric hydroxide dextran complex) has been used to establish systemic iron overload to study iron overload pathology in several organs.^24^,^26^ FeDex contains primarily ferric, and only a very low concentration of ferrous (0.8 %), iron.^27^ In this study, experimental mice were treated daily for 5 days each week with IP administration of 300 μL of 10 mg FeDex (Catalog No. D8517; Sigma-Aldrich Corp., St. Louis, MO, USA) diluted with PBS. Each experimental group consisted of three to five mice, and the treatment period was 2 or 4 weeks, with total iron loading of 100 mg or 200 mg, respectively. The control groups were given IP injections with the same volume of PBS for the same period of time as the corresponding experimental groups. There were three cohorts of WT mice in this study. The first cohort aged 2 months was treated with FeDex or PBS for 2 or 4 weeks, imaged and euthanized at 24 hours after the last injection, as shown in the schematic illustration (see Fig. 1A). The second cohort of WT mice aged 2 months was treated with FeDex or PBS for 4 weeks, imaged and euthanized at 9 months old, which was 6 months after the last injection (see Fig. 2A). The third cohort of WT mice aged 18 months was treated with FeDex or PBS for 4 weeks, imaged and euthanized at 22 months old, which was 3 months after the last injection (see Fig. 3A). Four-month-old mice with retina-specific *Hepc*KO (mRx-Cre^+^, Hepc^f/f^) and age-matched controls (mRx-Cre^+^, Hepc^+/+^), were IP injected with FeDex for 2 weeks, and their eyes were collected 24 hours after the last injection (see Fig. 5A).

### In Vivo Retina Imaging

Mice were anesthetized with an IP injection of (in mg/kg body weight) 80 ketamine (Par Pharmaceutical, Spring Valley, NY, USA), 10 xylazine (Lloyd Inc., Shenandoah, IA, USA) and 2 acepromazine (Boehringer Ingelheim Vetmedica, Inc. St. Joseph, MO, USA). Their pupils were dilated with 1% tropicamide (Akorn, Inc., Lake Forest, IL, USA). Once anesthetized adequately, mice were placed on a metal stage. Color and autofluorescence images were acquired using a fundus camera (Micron III; Phoenix Research Laboratories, Inc., Pleasanton, CA, USA), which uses a Xenon lamp producing white light. Autofluorescence imaging used a blue excitation (Semrock FF01–469/35) and a green emission (Semrock BLP01–488R) filter.

### Fixation of Eyes and Preparation of Eyecups

Eyes were enucleated immediately after euthanasia and fixed for 15 minutes in 4% paraformaldehyde and eyecups were created by removing the cornea and lens. Eyecups were dehydrated overnight in 30% sucrose and embedded in Tissue-Tek OCT (Sakura Finetek, Torrance, CA, USA).

### Immunofluorescence

Immunohistochemistry was performed on 10-μm-thick cryosections, as described previously.^1^ The following antibodies were used: rat anti-CD31 (1:100; Abcam, Cambridge, MA, USA), rabbit anti-light ferritin (E17) (1:2500; P. Arosio, University of Brescia, Italy), goat anti-albumin (1:200; Betyl Laboratories, Montgomery, TX, USA). Control sections were treated identically but with omission of primary antibody. Sections were analyzed by fluorescence microscopy with identical exposure parameters for comparison of experimentals with controls using Nikon Elements software (Melville, NY, USA).

### Dissection of Murine RPE and Retinas for RT-PCR

Mice were euthanized and eyes were immediately enucleated. Anterior segments were removed, and retinas were completely dissected away from the underlying RPE. Retinas were flash-frozen and stored at −80 °C. RPE cells were isolated from other ocular structures using enzymatic (dispase and hyaluronidase) digestion and mechanical dissection, as previously described.^10^

### Quantitative Real-Time PCR (qPCR)

RNA isolation was performed according to the manufacturer's protocol (RNeasy Kit; Qiagen, Valencia, CA, USA). cDNA was synthesized with reverse transcription reagents (Taqman; Applied Biosystems, Darmstadt, Germany) according to the manufacturer's protocol. The mRNA levels of iron regulatory genes and photoreceptor/RPE-specific genes were analyzed using qPCR as previously described.^8^ Gene expression assays (TaqMan; Applied Biosystems, Foster City, CA, USA) were used for qPCR analysis. Real-time RT-PCR was performed on a commercial sequence detection system (ABI Prism 7500; Applied Biosystems, Darmstadt, Germany). All reactions were performed in technical triplicates (*n* = 3–5 mice per genotype). Probes used were as follows: *Tfrc* (Mm00441941_m1), *Rho* (Mm00520345_m1), *Rpe65* (Mm00504133_m1), *Hamp* (Mm04231240_s1), *Opn1sw* (Mm00432058_m1), *Opn1mw* (Mm00433560_m1). *Gapdh* served as an internal control (4352932E).

### Quantitative Liver Iron Detection

Livers from experimental and matched control mice were frozen on dry ice. Dried tissue was digested overnight at 65°C in acid digest solution (0.1% trichloroacetic acid and 0.03mol/L HCl). After digestion, samples were centrifuged, and supernatant (20 μL) was added to 1 mL of chromogen reagent (2.25 mol/L sodium acetate pretreated with Chelex 100 [Bio-Rad, Hercules, CA, USA], 0.01% bathophenanthroline sulfonate [BPS], and 0.1% thioglycolic acid). The absorbances were read at 535 nm. Iron levels were calculated by comparing absorbances of tissue-chromogen samples to serial dilutions of iron standard (Sigma-Aldrich Corp.).

### Serum Iron Concentration and Transferrin (Tf) Saturation

Blood was collected from anesthetized animals by retro-orbital bleeding. Blood was collected in BD microtainer blood collection tubes (BD Biosciences, San Jose, CA, USA) and spun down for 30 minutes at 3000 rpm. Serum was collected and stored at −20°C. Serum Fe status was analyzed by quantifying total serum iron and transferrin saturation using an Iron/TIBC testing kit (Pointe Scientific, Inc., Canton, MI, USA). For some of the mice with serum iron overload, the Tf saturation calculation was over 100% due to the presence of non–transferrin-bound iron. If Tf saturation was more than 100%, it was recorded as 100% Tf saturation.

### Statistical Analysis

Mean ± SEM was calculated for each group. Student's two-group, two-tailed *t*-test was used for statistical analysis between two groups. For multiple comparisons, 1-way ANOVA with post hoc pairwise comparisons using the Bonferroni method was used. To determine the percentage of CD31+ blood vessels that were Ferritin-L+, a masked observer counted the number of CD31+ blood vessels and then determined how many of those blood vessels had colabeling with Ferritin-L in three stained retinal sections from each mouse (*n* = 3–5 mice/group). All statistical analyses were performed using GraphPad Prism 6.0 (San Diego, CA, USA).

## Results

### One Month of IP FeDex Treatment Does Not Increase NSR Iron But Causes Iron-Trapping in the Retinal Vasculature

To determine if the systemic iron overload mouse model was successfully established by IP FeDex, serum and liver were collected from each mouse in the first cohort. After 2 and 4 weeks of IP FeDex injection, serum iron levels increased by 21.2-fold and 63.0-fold, respectively, compared with PBS controls. In addition, there was a significant increase in serum iron levels after 4 weeks of FeDex injections compared with 2 weeks of FeDex injections (Fig. 1B). Serum Tf saturation was significantly increased after FeDex injection, with 100% serum Tf saturation in the 4 weeks of FeDex-injection group (Fig. 1C). The liver, as a representative organ indicating systemic iron levels, had markedly elevated iron levels in 2 and 4 weeks of FeDex-injection groups, by 365.4-fold and 405.4-fold, respectively, compared with PBS controls (Fig. 1D).

To determine how elevated systemic iron levels caused by IP FeDex injections influenced retinal iron levels, ferritin light chain (Ferritin-L) immunostaining was performed on cryosections of the retina. Ferritin-L, as a subunit of intracellular iron storage protein, ferritin, was used as an indirect but highly validated measure of iron accumulation in the retina.^8^,^9^,^20^,^28^ When intracellular labile iron levels increase, Ferritin-L transcription and translation are upregulated.^29^ After both 2 and 4 weeks of IP FeDex injection, the mice had Ferritin-L accumulation in the choroid, RPE, and CD31+ rVECs (Figs. 1E–H). The percentage of CD31+ VECs that were Ferritin-L+ was compared across the four groups. In the retinas of 2 weeks of FeDex-injection mice (Fig. 1F, arrows) and 4 weeks of FeDex-injection mice (Fig. 1H, arrows), there were significantly increased percentages of CD31+ rVECs that were colabeled with Ferritin-L compared with controls (Figs. 1E, 1G), as quantified in Figure 1I.

In vivo fundus imaging was used to investigate whether IP FeDex led to changes in retinal morphology. There was no evidence of hypopigmentation or autofluorescence in 4 weeks of FeDex-injection mice or matched controls (Fig. 1J, 1K).

To investigate NSR iron level changes and photoreceptor viability after 2 or 4 weeks of IP FeDex injection, NSR mRNA levels of transferrin receptor (*Tfrc*), hepcidin (*Hamp*), rhodopsin (*Rho*), cone opsin 1, short wave sensitive (*Opn1sw*), and cone opsin 1, medium wave sensitive (*Opn1mw*) were measured by qPCR (Fig. 1L). In addition to Ferritin-L immunolabeling, NSR iron levels were also determined using *Tfrc* mRNA levels, another validated measure of intracellular iron levels.^8^,^29^,^30^ *Tfrc* mRNA levels in the NSR did not change significantly across the four groups. *Hamp*, which encodes hepcidin, is expressed in multiple cell types in the retina, including photoreceptors, Müller cells, and RPE.^11^ There was no significant change in *Hamp* mRNA levels in the NSR of the mice with 2 weeks of FeDex injection. However, 4 weeks of FeDex injection induced a significant increase in *Hamp* mRNA levels. The relative mRNA levels of *Rho*, *Opn1sw*, and *Opn1mw* were quantified in the NSR to determine viability of rods and cones. The only significant, albeit small, magnitude changes were an increase in *Rho* mRNA after 2 weeks of FeDex injection and a decrease in *Opn1mw* after 4 weeks of FeDex injection.

To investigate the effect of FeDex on RPE iron levels and differentiation, relative mRNA levels of *Tfrc* and RPE (*Rpe65*) were measured (Fig. 1M). *Tfrc* mRNA levels in the RPE were significantly reduced by 68.9% after 2 weeks of FeDex injection, and reduced by 86.3% after 4 weeks of FeDex injection compared with age-matched PBS-injected controls, indicating RPE iron loading. *Rpe65* mRNA remained unchanged after 2 and 4 weeks of FeDex injection, consistent with the absence of RPE iron toxicity when loaded in this manner.

### Seven Months of Exposure to High Systemic Iron Through IP FeDex Injection Causes Mild Increase in NSR Iron, Hepcidin, and Mild Reduction in Cone Opsin

Four weeks of IP FeDex injection in 2-month-old WT mice led to iron accumulation in the RPE and rVECs, but did not increase NSR iron or induce photoreceptor degeneration. We then hypothesized that NSR iron accumulation and photoreceptor degeneration might occur with prolonged exposure to high systemic iron. To test this hypothesis, we injected 2-month-old WT mice with IP FeDex or PBS for 4 weeks and aged them to 9 months (Fig. 2A). At 9 months of age, the serum iron concentration of FeDex-injected mice increased by 31.3-fold compared with the PBS-injected mice (Fig. 2B). Serum Tf saturation in FeDex-injected mice increased compared with control, to an average of 93.5% (Fig. 2C). Liver iron concentration in the FeDex-injected mice was 178.7-fold higher compared with PBS-injected mice (Fig. 2D). Ferritin-L labeling was increased in the choroid, RPE, and CD31+ rVECs compared with control (arrows, Figs. 2E, 2F). The percentage of CD31+ rVECs that were Ferritin-L+ in the FeDex-injected mice was significantly higher compared with control (Fig. 2G). Although FeDex-injected mice had been exposed to high systemic iron for 7 months, there was no hypopigmentation or autofluorescence in the retina, similar to the phenotype of PBS-injected mice (Figs. 2H, 2I). *Tfrc* mRNA levels in the NSR collected 24 hours after 4 weeks of FeDex injection were unchanged compared with controls; however, *Tfrc* mRNA in the NSR collected 6 months later was moderately decreased by 33.0% compared with control (Fig. 2J). NSR *Hamp* was upregulated in FeDex-injected mice compared with control (Fig. 2J). *Rho* and *Opn1mw* mRNA levels in the NSR were not significantly different at 9 months, but *Opn1sw* mRNA was moderately reduced in FeDex-injected mice compared with control (Fig. 2J). In the RPE, *Tfrc* mRNA levels were significantly reduced by 83.0% in FeDex-injected mice compared with control, whereas *Rpe65* mRNA levels remained unchanged at 9 months (Fig. 2K).

### Four Months of Exposure to High Systemic Iron in 18-Month-Old Mice Causes a Mild Increase in NSR Iron and Hepcidin But No Photoreceptor Degeneration

Next, we hypothesized that photoreceptor degeneration might occur in aged mice with prolonged exposure to high systemic iron. We injected 18-month-old WT mice with IP FeDex or PBS for 4 weeks and aged them to 22 months (Fig. 3A). At 22 months, serum iron concentration of FeDex-injected mice increased by 14.9-fold compared with the PBS-injected mice (Fig. 3B). Tf saturation in the serum of FeDex-injected mice was increased compared to control, to an average of 98.3% (Fig. 3C). Liver iron concentration in the FeDex-injected mice was 294.8-fold higher compared with PBS-injected mice (Fig. 3D). Ferritin-L labeling was increased in the choroid, RPE, and CD31+ rVECs in the FeDex-injected aged mice compared with control (arrows, Figs. 3E, 3F). The percentage of CD31+ rVECs that were Ferritin-L+ in the FeDex-injected mice was significantly higher compared with control (Fig. 3G). There was no hypopigmentation or autofluorescence in the retina in the aged, FeDex-injected mice, similar to the phenotype of PBS-injected mice (Figs. 3H, 3I). *Tfrc* mRNA levels in the NSR were significantly decreased by 29.2% compared with control, indicating a mild increase in NSR iron (Fig. 3J). *Hamp* was significantly upregulated in the NSR of FeDex-injected mice compared with control. *Rho, Opn1sw*, and *Opn1mw* mRNA levels in the NSR were unchanged comparing FeDex and PBS-injected aged mice. In the RPE cells, *Tfrc* mRNA levels were reduced by 54.7% in FeDex-injected mice compared with control, whereas *Rpe65* mRNA levels were not significantly different (Fig. 3K).

### Iron Accumulation Within the rVECs Failed to Break Down the BRB

**Figure 4:**
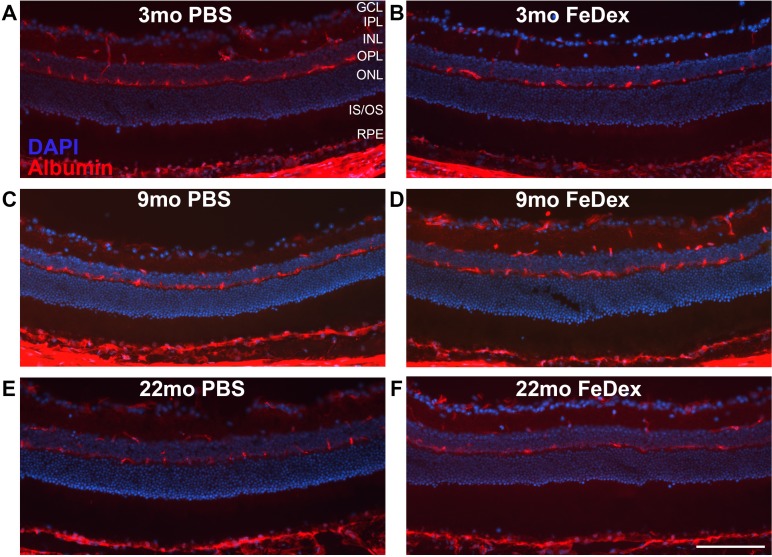
IP FeDex-induced iron accumulation within the rVECs fails to cause BRB disruption. Albumin immunostaining was performed to test for BRB integrity in the retinas from 3-month-old WT mice with FeDex injection (B), age-matched controls with PBS injection (A), 9-month-old WT mice with FeDex injection (D), age-matched control with PBS injection (C), 22-month-old WT mice with FeDex injection (F), and age-matched control with PBS injection (E). Scale bar: 50 μm. n = 3 per group.

To determine whether elevated iron levels in the rVECs of FeDex-injected mice resulted in BRB disruption, immunolabeling for the serum protein albumin was performed on all three cohorts, including 4 weeks of FeDex or PBS-injected mice analyzed at the age of 3 months, 9 months, and 22 months (Figs. 1A, 2A, 3A). When the BRB is intact, serum albumin cannot cross the BRB to enter the retina, and albumin immunolabeling should be found only within the blood vessels. Across the three cohorts, albumin was found to be confined to vessels in both FeDex and PBS-injected mice (Figs. 4A–F), indicating inner and outer BRB integrity in both young adults and aged mice after short- or long-term exposure to high systemic iron.

**Figure 5:**
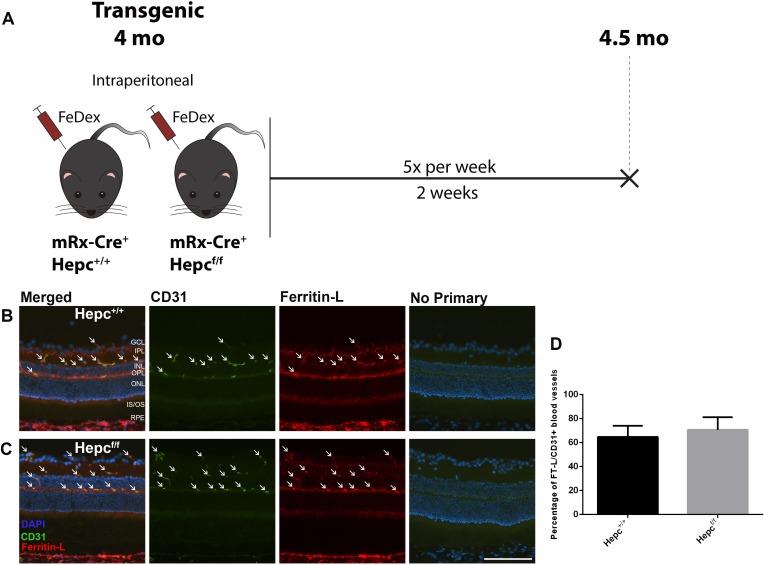
Iron-trapping is not induced by retinal hepcidin production. (A) Schematic illustration of the experimental design of the fourth cohort, 4-month-old transgenic mice were used. Both experimental (Hepc^f/f^, mRx-Cre^+^) and control mice (Hepc^+/+^, mRx-Cre^+^) were IP injected with FeDex for 2 weeks, and euthanized 24 hours after the last injection. (B and C) Ferritin-L immunostaining in the cryosections of the retinas. There was Ferritin-L labeling within the CD31+ rVECs (white arrows) in both experimental and control mice. (D) Quantification of the percentage of Ferritin-L+, CD31+ blood vessels in the retinas, indicating no difference between experimental and control mice. Statistical analyses were performed using Student's two-group, two-sided t-test. Error bars indicate ± SEM. Scale bar: 50 μm. n = 3 per group.

### Iron-Trapping Is Not Caused by Hepcidin Produced Within the NSR

Ferroportin (Fpn), localized to the abluminal membrane of rVECs,^20^ plays an important role in iron entry to the retina. Mice with *Fpn* deletion in the rVECs have Ferritin-L accumulation in the rVECs, indicating Fpn normally transfers iron from rVECs into the retina.^21^ A similar phenotype, iron-trapping within the rVECs, was found in all the FeDex-injected mice, which may occur due to Hepc-triggered Fpn degradation in the rVECs. Hepc is expressed in the NSR.^31^ Circulating Hepc does not have access to the extracellular domain of Fpn on the abluminal membrane of rVECs, therefore the Hepc-mediated Fpn degradation in the rVECs after IP FeDex injection must result from Hepc production by either the NSR or rVECs.

To test the hypothesis that rVEC iron-trapping resulted from NSR Hepc production after FeDex injection, eyes from retina-specific *Hepc*KO (mRx-Cre^+^, Hepc^f/f^) and age-matched control (mRx-Cre^+^, Hepc^+/+^), were collected after 2 weeks of IP FeDex injection (Fig. 5A). Ferritin-L accumulation was found within CD31+ rVECs in both retina-specific *Hepc*KO (Hepc^f/f^) and control (Hepc^+/+^) mice (arrows, Figs. 5B, 5C). The percentage of CD31+ rVECs that were Ferritin-L+ in the retina-specific *Hepc*KO (Hepc^f/f^) mice was not significantly different from Hepc^+/+^ (Fig. 5D). Importantly, mRx-Cre mice do not express Cre in rVECs, so rVECs from retina-specific *Hepc*KO mice still express Hepc.^25^

## Discussion

In this study, we analyzed the retinal phenotype observed after IP FeDex injection, a model of secondary iron overload. Acutely, the NSR does not accumulate iron. Chronically, after 9 months of systemic iron overload, it has only mildly elevated iron levels. There is no rod or RPE toxicity, and only mild cone toxicity, based on qPCR results. However, the rVECs become ferritin-positive after IP FeDex injection in all cohorts, suggesting that the inner BRB protects the NSR from fluctuations in systemic iron levels by trapping iron.

Although there was mild retinal iron accumulation, as indicated by decreased *Tfrc* mRNA levels, in the mice after 7 months of exposure to high systemic iron, and in the aged mice after 4 months of exposure to high systemic iron, there was no retinal degeneration except for a mild reduction in *Opn1sw* mRNA in the 9-month FeDex-injected mice. The somewhat increased susceptibility of cones compared with rods is consistent with observations in mice receiving intravitreal iron injection.^14^ The lack of NSR toxicity may result from the relatively small increase in NSR iron. Surprisingly, despite considerable elevations in RPE iron levels, indicated by marked reductions in *Tfrc* mRNA, there was no reduction in levels of the RPE-specific gene *Rpe65* and no RPE degeneration. These results suggest that PR and RPE degeneration in primary (hereditary) iron overload models results from excess iron entry into the NSR. Consistent with this assertion, the primary iron overload models, including *Cp/Heph* DKO, *Hepc*KO, Hepc-resistant *Fpn* knock-ins, and *Bmp6*KO have age-dependent iron accumulation in the NSR, followed by RPE hypertrophy/autofluorescence, and focal photoreceptor degeneration.^7^,^8^,^10^,^20^

Another important difference between the primary iron overload models and the IP FeDex model is that Ferritin-L levels increase in the rVECs only in the IP FeDex model. This finding suggests that iron is trapped in the rVECs only in IP FeDex model, possibly resulting from degradation of Fpn on the abluminal rVEC membrane.^20^ Previous work with cultured rVECs has shown that exposure to Hepc diminishes Fpn levels and iron export.^8^ Further, mice with conditional knockout of Fpn in rVECs have elevated rVEC and diminished NSR iron levels.^21^ However, we show herein that IP FeDex causes the same degree of rVEC iron accumulation in retina-specific *Hepc*KO mice compared to WT, indicating that Hepc produced by the NSR does not induce degradation of Fpn. The most likely remaining possibility is autocrine regulation of rVEC Fpn by Hepc. A similar autocrine Fpn regulation by Hepc has been reported in human monocytes, in cardiomyocytes, and in prostate cancer.^32^–^34^ In the primary iron overload mouse models resulting from systemic *Hepc*KO-, *Bmp6*KO-, and *Hepc-*resistant Fpn-knockin, all of which harbor defects in the Hepc/Fpn regulatory axis, this hypothesized autocrine loop would be impaired, consistent with the NSR iron accumulation, and lack of rVEC iron accumulation, observed in these models. The reasons for lack of apparent activation of an rVEC Hepc autocrine loop in *Cp/Heph* DKO and liver-specific *Hepc*KO will require better understanding of the mechanisms regulating this proposed rVEC autoregulation.

Overall, we find that the NSR is surprisingly unaffected by many months of exposure to extremely high levels of exogenous systemic iron. Increased iron in the rVECs suggests that these cells can shield the NSR from high serum iron levels. Blockade of iron export from the rVECs is the most likely explanation, resulting from Hepc-mediated degradation of Fpn, the only known cellular iron exporter, which localizes to the abluminal rVEC membrane. The lack of RPE degeneration despite iron accumulation in these cells suggests that iron is more toxic to the RPE when it enters the cells from the NSR. Impairment or bypass of the rVEC iron barrier has the potential to allow excess toxic iron to enter the retina, initiating or exacerbating vision loss.
